# Local-Field Corrections as a Regularization Method for the Spin-Boson Model

**DOI:** 10.1038/s41598-019-41303-0

**Published:** 2019-03-26

**Authors:** J. A. Crosse

**Affiliations:** 1 Division of Arts and Sciences, NYU Shanghai, 1555 Century Avenue, Pudong, Shanghai, 200122 China; 2 NYU-ECNU Institute of Physics at NYU Shanghai, 3663 Zhongshan Road North, Shanghai, 200062 China

## Abstract

The decoherence rate of a ‘central spin’ in a bosonic bath of magnetic fluctuations is computed using the spin-boson model. The magnetic fluctuations are treated in a fully quantum mechanical way by using the macroscopic quantum electrodynamics formalism and are expressed in terms of the classical electromagnetic Green’s function of the system. The resulting frequency integral formally diverges but it can be regularized by applying real-cavity, local-field corrections to the location of the ‘central spin’. This results in a cut-off function in terms of the magnetic permeability of the background material that leads to convergence at both high and low frequencies. This cut-off function appears naturally from the formalism and thus removes the need to rely on ad-hoc arguments to justify the form of the cut-off function. Furthermore, the magnetic permeability and the nature of interactions in quantum electrodynamics illuminate the connection between the two main models of ‘central spin’ decoherence, the spin-boson model and the spin-bath model, demonstrating how the two very different models are able to correctly model the same underlying physics.

## Introduction

Two-level quantum systems have been extensively studied owing to their potential application as qubits for quantum computing and quantum information processing^1,2^. The main property that facilitates these applications is the ability to generate and maintain a coherent superposition of the two eigenstates, a property that underpins many quantum information processing protocols. If the system is isolated the quantum coherence between the states can be maintained indefinitely. However, real quantum systems are inextricably linked to the environment. This coupling leads to ‘decoherence’ - the reduction of the pure superposition state to a mixed state of the two eigenstates. This is a major limiting factor in the realization of quantum information technology^3,4^.

Consider a spin-boson model where a two-level, ‘central spin’ is placed in a magnetic field, orientated in the *z*-direction, and coupled to an ‘environment’ consisting of longitudinal magnetic fluctuations described a bath of bosonic operators, ${\hat{b}}_{z}({\bf{r}},\omega )$. In the interaction picture, the ‘central spin’ evolves under the Hamiltonian

1 $${\hat{H}}_{I}(t)={\hat{S}}_{z}\,{\int }_{0}^{\infty }\,d\omega \,\lambda (\omega )\,[{\hat{b}}_{z}({{\bf{r}}}_{s},\omega ,t)+{\hat{b}}_{z}^{\dagger }({{\bf{r}}}_{s},\omega ,t)],$$

where ${\hat{S}}_{i}$ is the spin operator in the *i*-direction for the ‘central spin’ located at **r**_*s*_ and *λ*$(\omega )$ is the strength of the spin-bath coupling at frequency $\omega$. Under such an evolution the ‘central spin’ undergoes pure dephasing. The loss of coherence owing to the interaction with the environment is given by the expectation value of the coherence operator, ${\hat{S}}_{+}={\hat{S}}_{x}+i{\hat{S}}_{y}$,

2 $$L(t)=\langle {\hat{S}}_{+}\rangle =L(0){e}^{-\varphi (t)},$$

with

3 $$\varphi (t)={t}^{2}\,{\int }_{0}^{{\rm{\infty }}}\,d\omega \,{{\rm{s}}{\rm{i}}{\rm{n}}{\rm{c}}}^{2}(\omega t/2)\,\coth \,(\hslash \omega /2{k}_{b}T)J(\omega ).$$

Here, ${{\rm{sinc}}}^{2}(\omega t/2)$ is the free induction decay noise filter function and $\coth (\hslash \omega /2{k}_{b}T)$ is the thermal boson occupation number. $J(\omega )={\lambda }^{2}(\omega )$ is the spectral density, which completely describes the properties of the bosonic bath^4,5^.

The exact form of this spectral function is often introduced phenomenologically using physical arguments. A common choice is to assume a power law behaviour, $J(\omega )=A{\omega }^{s}$^3^. $s=1$ is referred to as an ‘ohmic’ bath whereas $0 < s < 1$ and $s > 1$ are referred to as ‘sub-ohmic’ and ‘super-ohmic’, respectively. Loosely speaking, a ‘super-ohmic’ spectral function corresponds to the case where the ‘central spin’ is under-damped by the environment whereas a ‘sub-ohmic’ spectral function corresponds to over-damping by the environment. The ‘ohmic’ spectral function corresponds to the critical transition between the two cases with the exact behaviour dependent on the bath-spin coupling^3^. For a power law spectrum it is clear that the frequency integral in Eq. (3) diverges. Hence, one needs to impose a frequency cutoff^3,5^, which is usually chosen to coincide with some physical parameter of the environment (e.g. the Debye frequency or the inverse of the Drude relaxation time). There have been a number of attempts to go beyond the phenomenological spectral densities that are often used to describe environmental fluctuations. For example, one area which has seen significant research in this direction is studies of photo-active biomolecules^6–9^ where the molecules and environments are usually too complex to be described accurately by a simple phenomenological model. In such cases sophisticated simulations or experimental data is required to find the form of the spectral density empirically. For simpler spin systems it would be advantageous to find a description that goes beyond the phenomenological but does not resort to highly complex first principle computer simulations or a require detailed experimental data.

In the following we revisit the spin-boson model but, instead of using a generic bosonic operator to describe the bosonic bath, we will use the macroscopic quantum electrodynamics formalism^10,11^ to exactly describe the magnetic fluctuations of the environment. This formalism allows one to express the bosonic bath in terms of the electromagnetic Green’s function of the system and results in a spectral function that can include the geometry of the surrounding material. Furthermore, by using the method of local-field corrections, one can regularize the divergent frequency integral in a more natural way.

Returning to Eq. (1), we wish to find a bosonic operator that correctly describes the magnetic fluctuations of the environment. To do this we start with Maxwell’s equations with a Langevin noise source that drives the classical electric and magnetic fields

4 $${\boldsymbol{\nabla }}\cdot {\bf{B}}({\bf{r}},\omega )=\mathrm{0,}$$

5 $${\boldsymbol{\nabla }}\times {\bf{E}}({\bf{r}},\omega )-i\omega {\bf{B}}({\bf{r}},\omega )={\bf{0}},$$

6 $${\boldsymbol{\nabla }}\cdot {\bf{D}}({\bf{r}},\omega )={\rho }_{N}({\bf{r}},\omega ),$$

7 $${\boldsymbol{\nabla }}\times {\bf{H}}({\bf{r}},\omega )+i\omega {\bf{D}}({\bf{r}},\omega )={{\bf{J}}}_{N}({\bf{r}},\omega ),$$

with the noise charge density and noise current density defined by $${\rho }_{N}({\bf{r}},\omega )={\boldsymbol{\nabla }}\cdot {{\bf{P}}}_{N}$$ and $${{\bf{J}}}_{N}({\bf{r}},\omega )=-\,i\omega {{\bf{P}}}_{N}({\bf{r}},\omega )+$$$${\boldsymbol{\nabla }}\times {{\bf{M}}}_{N}({\bf{r}},\omega )$$ respectively. Here, $${{\bf{P}}}_{N}({\bf{r}},\omega )$$ and $${{\bf{M}}}_{N}({\bf{r}},\omega )$$ are the noise polarization and noise magnetization fields that describe the electric and magnetic fluctuations within the material. To move to the quantum regime one needs to find the quantum bosonic operators which describe these noise fields. It is possible to canonically quantize these fields from first principles starting with the classical electromagnetic Lagrangian^12,13^. As a result one can write the noise fields in terms of two sets of canonical bosonic operators $${\hat{{\bf{f}}}}_{e}({\bf{r}},\omega )$$ and $${\hat{{\bf{f}}}}_{m}({\bf{r}},\omega )$$, which are associated with the electric and magnetic fluctuations respectively,

8 $${\hat{{\bf{P}}}}_{{\rm{N}}}({\bf{r}},\omega )=i\sqrt{\frac{\hslash {\varepsilon }_{0}}{\pi }{\rm{Im}}\varepsilon ({\bf{r}},\omega )}\,{\hat{{\bf{f}}}}_{e}({\bf{r}},\omega ),$$

9 $${\hat{{\bf{M}}}}_{{\rm{N}}}({\bf{r}},\omega )=i\sqrt{\frac{\hslash }{{\mu }_{0}\pi }\frac{{\rm{Im}}\mu ({\bf{r}},\omega )}{|\mu ({\bf{r}},\omega {)|}^{2}}}\,{\hat{{\bf{f}}}}_{m}({\bf{r}},\omega ).$$

Here, $\varepsilon ({\bf{r}},\omega )$ and $\mu ({\bf{r}},\omega )$ are the electric permittivity and magnetic permeability of the background medium, respectively, and ${\hat{{\bf{f}}}}_{\lambda }({\bf{r}},\omega )$ and ${\hat{{\bf{f}}}}_{\lambda }^{\dagger }({\bf{r}},\omega )$ obey the usual bosonic commutation relation

10 $$[{\hat{{\bf{f}}}}_{\lambda }({\bf{r}},\omega ),{\hat{{\bf{f}}}}_{\lambda ^{\prime} }^{\dagger }({\bf{r}}^{\prime} ,\omega ^{\prime} )]={\delta }_{\lambda \lambda ^{\prime} }{\boldsymbol{\delta }}({\bf{r}}-{\bf{r}}^{\prime} )\delta (\omega -\omega ^{\prime} \mathrm{).}$$

Resubstituting Eqs (8) and (9) in Maxwell’s equations, one can show that the magnetic field operator, which describes the magnetic fluctuations within the medium, is given by^10,11^

11 $${\hat{B}}_{z}({\bf{r}},\omega ,t)=\frac{{e}^{i\omega t}}{i\omega }\,\sum _{\lambda =e,m}\,\int \,{d}^{3}r^{\prime} \,\hat{z}\cdot {\boldsymbol{\nabla }}\times {{\boldsymbol{G}}}_{\lambda }({\bf{r}},{\bf{r}}{\boldsymbol{^{\prime} }},\omega )\cdot {\hat{{\bf{f}}}}_{\lambda }({\bf{r}}^{\prime} ,\omega \mathrm{).}$$

Here, the coefficients ${{\boldsymbol{G}}}_{\lambda }({\bf{r}},{\bf{r}}{\boldsymbol{^{\prime} }},\omega )$ are defined as

12 $${{\boldsymbol{G}}}_{e}({\bf{r}},{\bf{r}}^{\prime} ,\omega )=i\frac{{\omega }^{2}}{{c}^{2}}\sqrt{\frac{\hslash }{\pi {\varepsilon }_{0}}{\rm{Im}}\varepsilon ({\bf{r}}^{\prime} ,\omega )}\,{\boldsymbol{G}}({\bf{r}},{\bf{r}}^{\prime} ,\omega ),$$

13 $${{\boldsymbol{G}}}_{m}({\bf{r}},{\bf{r}}{\rm{^{\prime} }},\omega )=-\,i\frac{\omega }{c}\sqrt{\frac{\hslash }{\pi {\varepsilon }_{0}}\frac{{\rm{I}}{\rm{m}}\mu ({\bf{r}}{\rm{^{\prime} }},\omega )}{|\mu ({\bf{r}}{\rm{^{\prime} }},\omega ){|}^{2}}}[{\boldsymbol{G}}({\bf{r}},{\bf{r}}{\rm{^{\prime} }},\omega )\times \overleftarrow{{\rm{\nabla }}}{\rm{^{\prime} }}],$$

with the backward arrow referring to the fact that the operator acts on the right hand variable (here *r*′). The function, ${\boldsymbol{G}}({\bf{r}},{\bf{r}}^{\prime} ,\omega )$, is the electromagnetic Green’s function, which is the solution to the Helmholtz equation for a point source

14 $${\boldsymbol{\nabla }}\times \frac{1}{\mu ({\bf{r}},\omega )}{\boldsymbol{\nabla }}\times {\boldsymbol{G}}({\bf{r}},{\bf{r}}{\boldsymbol{^{\prime} }},\omega )-\frac{{\omega }^{2}}{{c}^{2}}\varepsilon ({\bf{r}},\omega ){\boldsymbol{G}}({\bf{r}},{\bf{r}}{\boldsymbol{^{\prime} }},\omega )=\delta ({\bf{r}}-{\bf{r}}{\boldsymbol{^{\prime} }}\mathrm{).}$$

Returning to Eq. (1), we can use Eq. (11) as the operator that describes the bosonic bath [i.e. $${\hat{B}}_{z}({\bf{r}},\omega ,t)=$$$${\hat{b}}_{z}({\bf{r}},\omega ,t)$$]. Now, instead of coupling our ‘central spin’ to a generic bosonic field we are able to couple it to a bosonic field that explicitly describes magnetic fluctuations within the material. However, this leads to a fundamental change in the structure of the Hamiltonian. Substituting Eq. (11) in to Eq. (1) leads to

15 $${\hat{H}}_{I}(t)={\hat{S}}_{z}\,{\int }_{0}^{\infty }\,d\omega \,\lambda (\omega )[\frac{{e}^{i\omega t}}{i\omega }\,\sum _{\lambda =e,m}\,\int \,{d}^{3}r^{\prime} \,\hat{z}\cdot {\boldsymbol{\nabla }}\times {{\boldsymbol{G}}}_{\lambda }({{\bf{r}}}_{s},{\bf{r}}{\boldsymbol{^{\prime} }},\omega )\cdot {\hat{{\bf{f}}}}_{\lambda }({\bf{r}}^{\prime} ,\omega )+{\rm{h}}.\,{\rm{c}}.].$$

Previously, one had the coupling constant, *λ*$(\omega )$, multiplied by the bosonic operator, ${\hat{b}}_{z}({{\bf{r}}}_{s},\omega ,t)$, with the phenomenological spectral density hidden in *λ*$(\omega )$. Now we have *λ*$(\omega )$ multiplied by the Green’s function ${\boldsymbol{\nabla }}\times {{\boldsymbol{G}}}_{\lambda }({{\bf{r}}}_{s},{\bf{r}}{\boldsymbol{^{\prime} }},\omega )$ multiplied by the the bosonic operator ${\hat{{\bf{f}}}}_{\lambda }({\bf{r}}^{\prime} ,\omega )$. Since the imaginary part of the Green’s function is related local density of states, the Green’s function is the equivalent of the spectral density, *J*$(\omega )$, but appears naturally out of the formalism rather than being introduced at a later stage. Now the coupling constant, *λ*$(\omega )$, is no longer related to the spectral density and merely represents the strength of the coupling of the ‘central spin’ to the magnetic fluctuations described by ${\boldsymbol{\nabla }}\times {{\boldsymbol{G}}}_{\lambda }({{\bf{r}}}_{s},{\bf{r}}{\boldsymbol{^{\prime} }},\omega )$ and hence can be treated as a constant, $\lambda (\omega )=\lambda$.

One can evaluate the expectation value of the coherence operator in the usual way (see Supplementary Information for more details). Expanding the magnetic field operators using Eq. (11), evalutating the time integrals, applying the thermal expectation values of the bosonic excitation operators

16 $$\langle {\hat{{\bf{f}}}}_{\lambda }({\bf{r}},\omega )\otimes {\hat{{\bf{f}}}}_{\lambda ^{\prime} }({\bf{r}}^{\prime} ,\omega ^{\prime} )\rangle ={\bf{0}},$$

17 $$\langle {\hat{{\bf{f}}}}_{\lambda }^{\dagger }({\bf{r}},\omega )\otimes {\hat{{\bf{f}}}}_{\lambda ^{\prime} }^{\dagger }({\bf{r}}^{\prime} ,\omega ^{\prime} )\rangle ={\bf{0}},$$

18 $$\langle {\hat{{\bf{f}}}}_{\lambda }^{\dagger }({\bf{r}},\omega )\otimes {\hat{{\bf{f}}}}_{\lambda ^{\prime} }({\bf{r}}^{\prime} ,\omega ^{\prime} )\rangle ={n}_{th}(\omega ){\delta }_{\lambda \lambda ^{\prime} }\delta ({\bf{r}}-{\bf{r}}^{\prime} )\delta (\omega -\omega ^{\prime} ){\mathbb{I}},$$

19 $$\langle {\hat{{\bf{f}}}}_{\lambda }({\bf{r}},\omega )\otimes {\hat{{\bf{f}}}}_{\lambda ^{\prime} }^{\dagger }({\bf{r}}^{\prime} ,\omega ^{\prime} )\rangle =[{n}_{th}(\omega )+\mathrm{1]}{\delta }_{\lambda \lambda ^{\prime} }\delta ({\bf{r}}-{\bf{r}}^{\prime} )\delta (\omega -\omega ^{\prime} ){\mathbb{I}},$$

where *n*_*th*_$(\omega )$ is the thermal photon number at temperature, *T*,

20 $${n}_{th}(\omega )=\frac{1}{{e}^{\hslash \omega /{k}_{B}T}-1},$$

and using the integral relation for the Green’s function

21 $$\sum _{\lambda =e,m}\,\int \,{d}^{3}s\,{{\boldsymbol{G}}}_{\lambda }({\bf{r}},{\bf{s}},\omega )\cdot {{\boldsymbol{G}}}_{\lambda }^{\dagger }({\bf{s}},{\bf{r}}^{\prime} ,\omega )=\frac{\hslash {\mu }_{0}}{\pi }{\omega }^{2}{\rm{Im}}\,{\boldsymbol{G}}({\bf{r}},{\bf{r}}^{\prime} ,\omega ),$$

one finds that $\varphi (t)$ reduces to

22 $$\begin{array}{ccc}\varphi (t) & = & {t}^{2}\,{\int }_{0}^{{\rm{\infty }}}\,d\omega \,{{\rm{s}}{\rm{i}}{\rm{n}}{\rm{c}}}^{2}(\omega t/2)\,\coth \,(\hslash \omega /2{k}_{b}T){\lambda }^{2}\frac{\hslash {\mu }_{0}}{\pi }\\  &  & \times \,\hat{z}\cdot \,{\boldsymbol{\nabla }}\times {\rm{I}}{\rm{m}}\,{\boldsymbol{G}}({{\bf{r}}}_{s},{{\bf{r}}}_{s},\omega )\times \overleftarrow{{\boldsymbol{\nabla }}}\cdot \hat{z}.\end{array}$$

One can see that Eq. (22) is identical to Eq. (3) except that the spectral function, *J*$(\omega )$, has been replaced by the double curl of the imaginary part of the Green’s function. The imaginary part of the Green’s function gives the local density of states, hence, in this formalism, the spectral function is given by the density of magnetic fluctuations at the location of the ‘central spin’. The usual approach to evaluating integrals of this type is to convert the frequency integral to a sum over the Matsubara frequencies^14^. However, in this case, this transformation is not possible because the ‘sinc’ function diverges at complex infinity and, as a result, it is not possible to close the contour integral in either half plane. Therefore, alternative methods for evaluating this integral must be found.

The effect of the quantum vacuum on the ‘central spin’ can be found by substituting the free space Green’s function in to Eq. (22). The free space Green’s function reads^10,11,15^

23 $${\boldsymbol{G}}({\bf{r}},{\bf{r}}^{\prime} ,\omega )=({\boldsymbol{\nabla }}\otimes {\boldsymbol{\nabla }}+\frac{\omega }{c}{n}^{2}(\omega ){\mathbb{I}})\frac{{e}^{i\omega n(\omega )|{\bf{r}}-{\bf{r}}^{\prime} |/c}}{4\pi {\omega }^{2}{n}^{2}(\omega )|{\bf{r}}-{\bf{r}}^{\prime} |/{c}^{2}},$$

with refractive index $n(\omega )=1$. In the following, we will also take the coupling of the ‘central spin’ to the magnetic field fluctuations to be equal to the gyromagnetic ratio, *γ*, given by $\lambda =\gamma =g{\mu }_{B}/\hslash$, where *g* is the landé *g*-factor and *μ*_*B*_ is the bohr magnaton. This leads to

24 $$\varphi (t)=\frac{\hslash {\mu }_{0}{\gamma }^{2}{t}^{2}}{6{\pi }^{2}{c}^{3}}\,{\int }_{0}^{\infty }\,d\omega \,{\omega }^{3}\,{{\rm{sinc}}}^{2}(\omega t\mathrm{/2})\,\coth \,(\hslash \omega \mathrm{/2}{k}_{b}T)\mathrm{.}$$

This integral is formally divergent and, in a similar way to other divergent couplings to the vacuum (such as the Lamb shift^16^), requires more sophisticated relativistic field theory methods to regularize. However, one should note that the coupling of a ‘central spin’ to the vacuum fluctuations is ‘super-ohmic’ and hence the ‘central spin’ is only weakly damped by the quantum vacuum.

In most cases of practical interest the ‘central spin’ will be embedded inside a medium. The Green’s function for homogeneous media is given by (23) with $n(\omega )\ne 1$. Unfortunately, the $\hat{z}\hat{z}$ component of the double curl of the Green’s function diverges when ${\bf{r}}\to {\bf{r}}^{\prime}$ unless the imaginary part of *n*$(\omega )$ vanishes, i.e. when there is no absorption within the material. As a non-zero imaginary part of the refractive index is necessary for the material response function to obey the Kramers-Krönig relations^17^, which are themselves a result of causality, it is not possible for a real material to be absorption free over the whole frequency range. Hence, the Green’s function for homogeneous media leads to divergent results and a further regularization step is needed.

**Figure 1:**
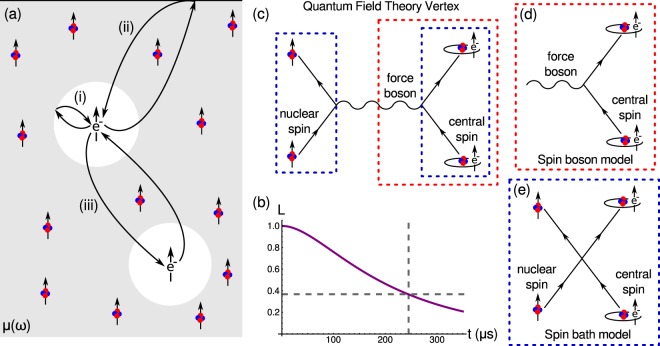
The interaction of a ‘central spin’ with magnetic fluctuations created by a nuclear spin bath. (**a**) Schematic of the local-field correction method (i) the local field correction is computed by considering electromagnetic waves reflected from the wall of a spherical cavity. The Green’s function method also allows one to compute the contribution from (ii) inhomogeneities and (iii) specific spins by considering the appropriate reflection terms from the Green’s function. (**b**) The coherence of a ‘central spin’ with time for the parameters given in the main text. The grey dashed lines show the coherence time of *t*_*coh*_ ≈ 245 *μ*s which is taken to be the time when the coherence has dropped to *L* = *e*^−1^ = 36.8%. The quantum field description (**c**) of an interaction between two spins is described in terms of the exchange of a force boson. The spin-boson model (**d**) only considers the part in the red dashed box whereas the spin-bath model (**e**) only considers the part in the blue dashed box.

The diverging Green’s function and frequency integral can both be regularized by performing local-field corrections on the magnetic field fluctuations at the location of the ‘central spin’. Local-field corrections aim to provide more accurate expressions for the macroscopic polarization and magnetization fields within a medium by including shielding or local enhancement effects from the matter itself. The standard approach to performing these corrections is to use a real-cavity model^18^ where we assume that the ‘central spin’ lies at the centre (${\bf{r}}=0$) of a spherical cavity [c.f. Fig. 1a(i)]. This model is the quantum mechanical equivalent of the Onsager model^19,20^ that is often used in physical chemistry to describe the a polarizable molecule in a dielectric background^21,22^ and results in a correction to the polarizability that is a function of the permittivity of the background medium. The technique has been used to study an number of quantum optical processes in media such as spontaneous decay rates inside dielectric media^23,24^ and to compute corrections to the Van der Waals^25^, Casimir-Polder^26^ and nonlinear ${\chi }^{(2)}$^27^ interactions. The technique can be applied, not only to dielectric backgrounds, but to magneto-dielectric backgrounds as well with one finding a correction to the polarizability that is both a function of the permittivity and permeability of the background medium^25^.

The correction to the local field by the surrounding matter is found by computing the reflection of the electromagnetic field at the cavity wall^24,25^. The Green’s function for a spherical cavity reads

25 $$\begin{array}{rcl}{\boldsymbol{G}}({\bf{r}},{\bf{r}}^{\prime} ,\omega ) & = & \frac{ik}{4\pi }\,\sum _{n\in e,o}\,\sum _{l=1}^{\infty }\,\sum _{m=0}^{l}\,\mathrm{(2}-{\delta }_{l0})\frac{2l+1}{l(l+\mathrm{1)}}\frac{(l-m)!}{(l+m)!}\\  &  & \times \,[{R}_{TE}(\omega ){{\boldsymbol{M}}}_{mln}(k,{\bf{r}})\otimes {{\boldsymbol{M}}}_{mln}(k,{\bf{r}}^{\prime} )\\  &  & +\,{R}_{TM}(\omega ){{\boldsymbol{N}}}_{mln}(k,{\bf{r}})\otimes {{\boldsymbol{N}}}_{mln}(k,{\bf{r}}^{\prime} )],\end{array}$$

where *R*_*TE*_ and *R*_*TM*_ are the reflection coefficients for the *TE* and *TM* polarized waves respectively and the ${{\boldsymbol{M}}}_{mln}(k,{\bf{r}})$ and ${{\boldsymbol{N}}}_{m\mathrm{ln}}(k,{\bf{r}})$ dyads are given by

26 $$\begin{array}{rcl}{{\boldsymbol{M}}}_{ml({}^{e}o)}(k,{\bf{r}}) & = & \mp \frac{m}{\sin \,\theta }{j}_{l}(kr){P}_{l}^{m}(\cos \,\theta )(\begin{array}{c}\sin \,m\varphi \\ \cos \,m\varphi \end{array}){\underline{e}}_{\theta }\\  &  & -\,{j}_{l}(kr)\frac{d{P}_{l}^{m}(\cos \,\theta )}{d\theta }(\begin{array}{c}\cos \,m\varphi \\ \sin \,m\varphi \end{array}){\underline{e}}_{\varphi },\end{array}$$

27 $$\begin{array}{rcl}{{\boldsymbol{N}}}_{ml({}^{e}o)}(k,{\bf{r}}) & = & \frac{l(l+\mathrm{1)}}{kr}\,{j}_{l}(kr){P}_{l}^{m}(\cos \,\theta )(\begin{array}{c}\cos \,m\varphi \\ \sin \,m\varphi \end{array}){\underline{e}}_{r}\\  &  & +\,\frac{1}{kr}\frac{d}{dr}[r{j}_{l}(kr)]\,[\frac{d{P}_{l}^{m}(\cos \,\theta )}{d\theta }(\begin{array}{c}\cos \,m\varphi \\ \sin \,m\varphi \end{array}){\underline{e}}_{\theta }\\  &  & \mp \,\frac{m}{\sin \,\theta }{P}_{l}^{m}(\cos \,\theta )(\begin{array}{c}\sin \,m\varphi \\ \cos \,m\varphi \end{array}){\underline{e}}_{\varphi }],\end{array}$$

where *j*_*l*_(*x*) are spherical Bessel functions of the first kind and ${P}_{l}^{m}(x)$ are the associated Legendre polynomials^10,11,15^. By computing the curl of the individual dyads and then taking ${\bf{r}},{\bf{r}}^{\prime} \to 0$ one finds that the only contribution is from $l=1$ and $m=0$. Hence, the *TM* mode vanishes and only the *TE* mode contributes. Thus, the Green’s function reduces to

28 $${\hat{z}\cdot {\boldsymbol{\nabla }}\times {\boldsymbol{G}}({\bf{r}},{\bf{r}}^{\prime} ,\omega )\times \overleftarrow{{\boldsymbol{\nabla }}}\cdot \hat{z}|}_{{\bf{r}},{\bf{r}}^{\prime} \to 0}=i\frac{{\omega }^{3}}{6\pi {c}^{3}}{R}_{TE}(\omega \mathrm{).}$$

The reflection of the *TE* modes at the cavity interface can be described in terms of the Mie scattering coefficient

29 $${R}_{TE}(\omega )=\frac{{h}_{1}^{\mathrm{(1)}}({z}_{0})[z{h}_{1}^{\mathrm{(1)}}(z)]^{\prime} -\mu (\omega ){h}_{1}^{\mathrm{(1)}}(z)[{z}_{0}{h}_{1}^{\mathrm{(1)}}({z}_{0})]^{\prime} }{\mu (\omega ){h}_{1}^{\mathrm{(1)}}(z)[{z}_{0}{j}_{1}^{\mathrm{(1)}}({z}_{0})]^{\prime} -{j}_{1}^{\mathrm{(1)}}({z}_{0})[z{h}_{1}^{\mathrm{(1)}}(z)]^{\prime} }$$

where ${z}_{0}=\omega {R}_{c}/c$ and $z=n(\omega )\omega {R}_{c}/c$, with *R*_*c*_ the radius of the cavity, and *j*_1_(*z*) and *h*_1_(*z*), respectively, the spherical Bessel and Hankel functions of the first kind for $l=1$,

30 $${j}_{1}(z)=\frac{\sin (z)}{{z}^{2}}-\frac{\cos (z)}{z},$$

31 $${h}_{1}(z)=(\frac{1}{z}+\frac{i}{{z}^{2}}){e}^{iz}.$$

We will assume that *R*_*c*_ is small compared to the main wavelengths associated with the decoherence process (in fact the free induction decay filter is strongly peaked around $\omega =0$ so the main wavelength → ∞) and we expand ${R}_{TE}(\omega )$ in powers of $\omega {R}_{c}/c$

32 $$\begin{array}{rcl}{R}_{TE}(\omega ) & = & \frac{3\mu (\omega )-3}{[2\mu (\omega )+1]}\frac{i{c}^{3}}{{\omega }^{3}{R}_{c}^{3}}\\  &  & +\,\frac{9}{5}\{\frac{\mu {(\omega )}^{2}[5\varepsilon (\omega )-1]-3\mu (\omega )-1]}{{[2\mu (\omega )+1]}^{2}}\}\frac{ic}{\omega {R}_{c}}\\  &  & -\,9\frac{\mu {(\omega )}^{5/2}\varepsilon {(\omega )}^{3/2}}{{[2\mu (\omega )+1]}^{2}}+1+{\mathscr{O}}(\frac{\omega {R}_{c}}{c}).\end{array}$$

By taking the reflection coefficient to leading order, computing the imaginary part of Eq. (28) and substituting the result into Eq. (22), one finds that

33 $$\begin{array}{rcl}\varphi (t) & = & \frac{3\hslash {\mu }_{0}{\gamma }^{2}{t}^{2}}{2{\pi }^{2}{R}_{c}^{3}}\,{\int }_{0}^{\infty }\,d\omega \,{{\rm{sinc}}}^{2}(\omega t\mathrm{/2})\,\coth \,(\hslash \omega \mathrm{/2}{k}_{b}T)\\  &  & \times \,\frac{-{\rm{Im}}\,\mu (\omega )}{\mathrm{4[}{\rm{Im}}\,\mu (\omega {)]}^{2}+{\mathrm{[1}+2{\rm{Re}}\mu (\omega )]}^{2}},\end{array}$$

where again we have taken *λ* to be equal to the gyromagnetic ratio *γ*. If the permeability, *μ*$(\omega )$, follows a functional behaviour similar to the Drude or Drude-Lorenz models then as $\omega \to 0$, the imaginary part vanishes whereas the real part tends to some finite value. Furthermore, high frequency transparency means that as $\omega \to \infty$, the imaginary part vanishes whereas the real part tends to unity. Hence, the integrand is regularized in both the high and low frequency limits and the integral can be evaluated.

As an example, one can use the common frequency dependent paramagnetic permeability model^28^

34 $$\mu (\omega )=\frac{\mu (0)-\mu (\infty )}{1+i\omega \tau }+\mu (\infty ),$$

where *μ*(0) and *μ*$(\infty )$ are the constant permeabilities at $\omega \to 0$ and $\omega \to \infty$, respectively, and *τ* is the magnetization relaxation time (i.e. the time it takes the background spins to de-align when the magnetic field is switched off). With this permeability model we find the material factor in Eq. (33) becomes

35 $$\frac{-{\rm{Im}}\,\mu (\omega )}{4{[{\rm{Im}}\mu (\omega )]}^{2}+{[1+2{\rm{Re}}\mu (\omega )]}^{2}}=\frac{[\mu (0)-\mu (\infty )]\omega \tau }{{[1+2\mu (0)]}^{2}+{[1+2\mu (\infty )]}^{2}{\omega }^{2}{\tau }^{2}}.$$

This expression clearly does not have the simple power law functionality that is usually assumed for spectral functions. However, it is approximately ‘ohmic’ at low frequencies and vanishes as $\omega \to 0$ and $\omega \to \infty$. Furthermore, it is functionally similar to the phenomenological ‘soft cutoff’ functions that have previously been used to regularize this type of integral^3,5^. Thus, we can see that the precise nature of the phenomenological soft cutoff required to regularize the integral can be derived directly from the form of the permeability function.

It is worth noting that the frequency dependence of Green’s function is significantly different before and after regularization. This is due to the nature of the fluctuations that the ‘central spin’ is interacting with in each case. In the vacuum, the ‘central spin’ couples to the vacuum fluctuations and the spectral density displays a ${\omega }^{3}$ dependence. In a homogeneous media before regularization and (necessarily) in the absence of absorption the spectral density displays a ${\omega }^{3}\,{\rm{Re}}\,{[n(\omega )]}^{3}$ dependence. Essentially, one is still coupling to ‘vacuum fluctuations’, however, these fluctuations are now scaled by the response of the medium - the refractive index merely adds a multiplicative factor to the vacuum result. By performing the regularization step one changes the coupling of the ‘central spin’ from a coupling to the fluctuations of the vacuum to a coupling to the fluctuations of the medium. In an absorbing medium the fluctuation dissipation theorem^29,30^ states that any process that dissipates energy will lead to background fluctuations. In this case, absorption in the material leads to fluctuations in the magnetic response of the medium. It is to these fluctuations that the ‘central spin’ couples to after regularization. This can be seen from Eq. (35), which is a function of the the magnetization relaxation time $\tau$, which itself is a measure of the absorption properties of the surrounding material.

A specific physical spin system that has been studied extensively is the spin decoherence of phosphorous donors in silicon. In this system, the main decoherence process is driven by the nuclear spins of the ^29^Si isoptope (the other two stable isotopes, ^28^Si and ^30^Si have no nuclear spin) which is present with an abundance of 4.7% in natural silicon. Exact numbers for the permeability contribution of these spins alone (without the diamagnetic contribution from the silicon atoms) are hard to find but we can make reasonable order of magnitude estimates based on the properties of the spins themselves. The susceptibilities unpaired electrons in paramagnetic atoms is on the order of $\chi \approx 1\times {10}^{-5}$ to $2\times {10}^{-5}$. However, the coupling of electron spins to magnetic fields is much stronger than that of nuclear spins and can be quantified by the square of the gyromagnetic ratio. The gyromagnetic ratio for an electron is $\gamma =1.76\times {10}^{11}\,{\rm{rads}}\,{{\rm{s}}}^{-1}\,{{\rm{T}}}^{-1}$ whereas the gyromagnetic ratio for ^29^Si nuclear spins is three orders of magnitude smaller, ${\gamma }_{I}=5.32\times {10}^{7}\,{\rm{rads}}\,{{\rm{s}}}^{-1}\,{{\rm{T}}}^{-1}$. Scaling the paramagnetic susceptibility with regard to the weaker coupling and lower concentration of nuclear spins leads to an estimate the nuclear susceptibility on the order of ${\chi }_{I}\approx 9\times {10}^{-14}$. The spin relaxation time is estimated to be $\tau \approx 6\times {10}^{-5}\,{\rm{s}}$ from the 2nd moment (variance) of the observed linewidth^31^. The last parameter is the characteristic radius, *R*_*c*_, which we take to be half the average separation between nuclear spins (i.e. the average distance from the ‘central’ spin to the nearest nuclear spin). The atomic density of silicon is ${\rho }_{N}\approx 5\times {10}^{28}\,{{\rm{m}}}^{-3}$ of which only 4.7% are ^29^Si, hence the characteristic radius is estimated to be ${R}_{c}\approx 7\times {10}^{-10}\,{\rm{m}}$. These parameters lead to a coherence time of ${t}_{coh}\approx 245$ *μ*s at 6 *K*, which is the same order of magnitude as the experimentally measured spin decoherence times in this system^32^. The full curve is shown in Fig. 1(b).

Clearly, for a quantitatively more precise result the permeability function and related parameters needs to be computed or measured more accurately. To do this the response of the appropriate spin species to an applied magnetic field needs to be found. Hence, one can immediately see the connection between the two main models of decoherence; the spin-boson model, as described here, which models the effect of a bosonic environment on the ‘central spin’ and the spin-bath model, which models the effect individual spins on the the ‘central spin’. From the results presented here, we can see that the bosonic environment is generated by magnetic fluctuations described by a permeability, which itself is a macroscopic description of how the individual spins of the background spin-bath behave in the presence of an external magnetic field. From a quantum field theory perspective such a link is not surprising as any interaction between particles is mediated by a force boson - a photon in the case of electromagnetism [c.f. Fig. 1(c)]. The spin-boson model describes the environment in terms of the force bosons whilst neglecting their fermionic source [c.f. Fig. 1(d)]. The spin-bath model describes the environment in terms of fermionic sources whilst neglecting the force boson [c.f. Fig. 1(e)]. The functional link between the two models (in this case) is the macroscopic permeability which describes the force bosons for a given set of fermionic sources.

Developing accurate permeability models is itself non-trivial. Traditional semi-classical methods look at the response of single quantum particles to a classical field and then obtain a macroscopic result by simply multiplying the single particle result by the particle number density^33^. However, spin-bath studies of ‘central spin’ decoherence have shown that intra-bath correlations can also contribute^34^. Thus, fully quantum methods for computing the magnetic susceptibilities are potentially required for accurate calculations of the coherence time. However, these fully quantum methods in conjunction with the results presented here would constitute a precise analytical method for computing decoherence times of two level systems.

We conclude by mentioning in passing that, by using the result of Refs^24,25^, the Green’s function approach allows one to compute the effect of material inhomogeneities (such as surfaces, interfaces and cavities [c.f. Fig. 1a(ii)]) on the ‘central spin’ decoherence or the interaction with specific spins [c.f. Fig. 1a(iii)] simply by including the appropriate Green’s function. Hence, the formalism presented here allows for a more versatile description the interaction of a ‘central spin’ with bosonic or spin-bath environments.

## Supplementary information

SUPPLEMENTARY MATERIAL for Local-Field Corrections as a Regularization Method for the Spin-Boson Model
