# Supplementary material for: Local-Field Corrections as a Regularization Method for the Spin-Boson Model
Source: Sci Rep. 2019 Mar 26;9:5216. doi: 10.1038/s41598-019-41303-0 (PMC6435645; doi:10.1038/s41598-019-41303-0)
Supplement: Supplementary file 1 — SUPPLEMENTARY MATERIAL for Local-Field Corrections as a Regularization Method for the Spin-Boson Model [file 41598_2019_41303_MOESM1_ESM.pdf]

SUPPLEMENTARY MATERIAL for Local-Field Corrections as a Regularization Method for the Spin-Boson Model

J. A. Crosse[1,2,*]

[1]New York University Shanghai, 1555 Century Avenue, Pudong, Shanghai, 200122, China.
[2]NYU-ECNU Institute of Physics at NYU Shanghai, 3663 Zhongshan Road North, Shanghai, 200062, China.
[*]jac32@nyu.edu

Coherence

The interaction Hamiltonian for a 'central spin' in a magnetic field orientated in the z-direction reads

$$\hat{H}_I = \hat{b}_z(\mathbf{r}_s,t)\hat{S}_z. \tag{1}$$

Here $\hat{S}_z$ is the spin operator for the 'central spin' and $\hat{b}_z(\mathbf{r}_s,t)$ is the operator for the bosonic magnetic fluctuations of the environment at $\mathbf{r}_s$, the location of the 'central spin' (and includes a frequency dependent coupling parameter). The coherence of the 'central spin' can be found from the expectation value of the coherence operator, $\hat{S}_+ = \hat{S}_x + i\hat{S}_y$, which is found to be

$$\begin{aligned}
\langle \hat{S}_+ \rangle &= \mathrm{Tr}\left\{ \hat{S}_+ \exp\left[-\frac{i}{\hbar}\int \hat{b}_z(\mathbf{r}_s,t)\hat{S}_z dt \right] \hat{\rho}_I^{(0)} \exp\left[\frac{i}{\hbar}\int \hat{b}_z(\mathbf{r}_s,t)\hat{S}_z dt \right] \right\}, \\
&= \mathrm{Tr}\left\{ \exp\left[\frac{i}{2}\int \hat{b}_z(\mathbf{r}_s,t) dt \right] \hat{S}_+(t)\, \hat{\rho}_I^{(0)} \exp\left[\frac{i}{\hbar}\int \hat{b}_z(\mathbf{r}_s,t)\hat{S}_z dt \right] \right\}, \\
&= \mathrm{Tr}\left\{ \hat{S}_+(t)\, \hat{\rho}_I^{(0)} \exp\left[\frac{i}{2}\int \hat{b}_z(\mathbf{r}_s,t)\hat{\sigma}_z dt \right] \exp\left[\frac{i}{2}\int \hat{b}_z(\mathbf{r}_s,t) dt \right] \right\}.
\end{aligned}$$

Using the Baker-Campbell-Hausdorff formula one arrives at

$$\begin{aligned}
&= \mathrm{Tr}\left\{ \hat{S}_+(t)\, \hat{\rho}_I^{(0)} \exp\left[i\int \hat{b}_z(\mathbf{r}_s,t)\hat{\sigma}_\uparrow dt \right] \right\}, \\
&= \frac{\hbar}{2}\rho_{du}^{(0)} \left\langle \exp\left[i\int \hat{b}_z(\mathbf{r}_s,t) dt \right] \right\rangle,
\end{aligned} \tag{2}$$

where $\hat{\sigma}_\uparrow = \mathrm{diag}[1,0]$ is the projection operator on to the up spin state. As the magnetic field is written as a linear combination of bosonic creation and annihilation operators, the expectation value of the exponential factor is given by

$$\langle e^{\phi} \rangle = e^{\langle \phi^2 \rangle /2}. \tag{3}$$

Hence the expectation value of the field operators becomes

$$\langle \hat{S}_+ \rangle = \rho_{du}^{(0)} \left\langle \exp\left[i\int \hat{b}_z(\mathbf{r}_s,t) dt \right] \right\rangle = \rho_{du}^{(0)} \exp\left[-\frac{1}{2}\int dt' \int dt'' \left\langle \hat{b}_z(\mathbf{r}_s,t')\hat{b}_z(\mathbf{r}_s,t'') \right\rangle \right]. \tag{4}$$

In the following we will expand the magnetic field as

$$\hat{b}(\mathbf{r},t) = \int_0^\infty d\omega\, \lambda(\omega) \left[\hat{b}(\mathbf{r},\omega,t) + \hat{b}^\dagger(\mathbf{r},\omega,t) \right], \tag{5}$$

with $\lambda(\omega)$ a the frequency dependent coupling parameter and

$$\hat{b}(\mathbf{r},\omega,t) = \frac{1}{i\omega}\sum_{\lambda=e,m} \int d^3r'\, \boldsymbol{\nabla} \times \boldsymbol{G}_\lambda(\mathbf{r},\mathbf{r}',\omega) \cdot \hat{\mathbf{f}}(\mathbf{r}',\omega,t), \tag{6}$$

where $\hat{\mathbf{f}}(\mathbf{r},\omega,t)$ and $\hat{\mathbf{f}}^{\dagger}(\mathbf{r},\omega,t)$ are bosonic annihilation and creation operators that obey the usual commutation relation

$$\left[\hat{\mathbf{f}}_{\lambda}(\mathbf{r},\omega),\hat{\mathbf{f}}^{\dagger}_{\lambda'}(\mathbf{r}',\omega')\right]=\delta_{\lambda\lambda'}\boldsymbol{\delta}(\mathbf{r}-\mathbf{r}')\delta(\omega-\omega'). \tag{7}$$

The coefficients $\boldsymbol{G}_{\lambda}(\mathbf{r},\mathbf{r}',\omega)$ are given by

$$\boldsymbol{G}_{e}(\mathbf{r},\mathbf{r}',\omega)=i\frac{\omega^2}{c^2}\sqrt{\frac{\hbar}{\pi\varepsilon_0}\mathrm{Im}\varepsilon(\mathbf{r}',\omega)}\boldsymbol{G}(\mathbf{r},\mathbf{r}',\omega), \tag{8}$$

$$\boldsymbol{G}_{m}(\mathbf{r},\mathbf{r}',\omega)=-i\frac{\omega}{c}\sqrt{\frac{\hbar}{\pi\varepsilon_0}\frac{\mathrm{Im}\mu(\mathbf{r}',\omega)}{|\mu(\mathbf{r}',\omega)|^2}}\left[\boldsymbol{G}(\mathbf{r},\mathbf{r}',\omega)\times\overleftarrow{\boldsymbol{\nabla}}'\right], \tag{9}$$

with the backward arrow referring to the fact that the operator acts on the right hand variable (here r'). The function, $\boldsymbol{G}(\mathbf{r},\mathbf{r}',\omega)$, is the electromagnetic Green's function, which is the solution to the Helmholtz equation for a point source

$$\boldsymbol{\nabla}\times\boldsymbol{\mu}^{-1}(\mathbf{r},\omega)\boldsymbol{\nabla}\times\boldsymbol{G}(\mathbf{r},\mathbf{r}',\omega)-\frac{\omega^2}{c^2}\boldsymbol{\varepsilon}(\mathbf{r},\omega)\boldsymbol{G}(\mathbf{r},\mathbf{r}',\omega)=\delta(\mathbf{r}-\mathbf{r}'). \tag{10}$$

Here, $\boldsymbol{\varepsilon}(\mathbf{r},\omega)$ and $\boldsymbol{\mu}(\mathbf{r},\omega)$ are the electric permittivity and magnetic permeability tensors respectively.

The field expectation value can be computed as follows

$$\begin{aligned}\left\langle\hat{b}_z(\mathbf{r}_s,t')\hat{b}_z(\mathbf{r}_s,t'')\right\rangle &= \int_0^\infty d\omega\int_0^\infty d\omega'\left\langle\left[\hat{b}_z(\mathbf{r}_s,\omega,t')+\hat{b}_z^{\dagger}(\mathbf{r}_s,\omega,t')\right]\left[\hat{b}_z(\mathbf{r}_s,\omega',t'')+\hat{b}_z^{\dagger}(\mathbf{r}_s,\omega',t'')\right]\right\rangle,\\
&=\sum_{\lambda=e,m}\sum_{\lambda'=e,m}\int_0^\infty d\omega\int_0^\infty d\omega'\,\frac{\lambda(\omega)}{\omega}\frac{\lambda(\omega')}{\omega'}\int d^3r'\int d^3r''\\
&\quad\left[\hat{z}\cdot\boldsymbol{\nabla}\times\boldsymbol{G}_{\lambda}(\mathbf{r}_s,\mathbf{r}',\omega)\otimes\boldsymbol{G}^{\dagger}_{\lambda'}(\mathbf{r}'',\mathbf{r}_s,\omega')\times\overleftarrow{\boldsymbol{\nabla}}\cdot\hat{z}:\left\langle\hat{\mathbf{f}}(\mathbf{r}',\omega)\otimes\hat{\mathbf{f}}^{\dagger}(\mathbf{r}'',\omega')\right\rangle_T e^{-i\omega t'}e^{i\omega' t''}\right.\\
&\quad\left.+\hat{z}\cdot\boldsymbol{\nabla}\times\boldsymbol{G}^{\dagger}_{\lambda}(\mathbf{r}_s,\mathbf{r}',\omega)\otimes\boldsymbol{G}_{\lambda'}(\mathbf{r}'',\mathbf{r}_s,\omega')\times\overleftarrow{\boldsymbol{\nabla}}\cdot\hat{z}:\left\langle\hat{\mathbf{f}}^{\dagger}(\mathbf{r}',\omega)\otimes\hat{\mathbf{f}}(\mathbf{r}'',\omega')\right\rangle_T e^{i\omega t'}e^{-i\omega' t''}\right],\end{aligned} \tag{11}$$

where we have assumed that the bosonic operators evolve freely and noted that the expectation value of the other bilinear combinations of the bosonic operators vanish upon taking expectation values. Using the remaining non-vanishing thermal expectation values

$$\langle\hat{\mathbf{f}}^{\dagger}_{\lambda}(\mathbf{r},\omega)\otimes\hat{\mathbf{f}}_{\lambda'}(\mathbf{r}',\omega')\rangle_T=n_{th}(\omega)\delta_{\lambda\lambda'}\delta(\mathbf{r}-\mathbf{r}')\delta(\omega-\omega')\mathbb{I}, \tag{12}$$

$$\langle\hat{\mathbf{f}}_{\lambda}(\mathbf{r},\omega)\otimes\hat{\mathbf{f}}^{\dagger}_{\lambda'}(\mathbf{r}',\omega')\rangle_T=[n_{th}(\omega)+1]\delta_{\lambda\lambda'}\delta(\mathbf{r}-\mathbf{r}')\delta(\omega-\omega')\mathbb{I}, \tag{13}$$

where $n_{th}(\omega)$ is the thermal photon number at temperature, T,

$$n_{th}(\omega)=\frac{1}{e^{\hbar\omega/k_BT}-1}, \tag{14}$$

(k_B is Boltzmann's constant) and the integral relation for the Green's function

$$\sum_{\lambda=e,m}\int d^3s\,\boldsymbol{G}_{\lambda}(\mathbf{r},\mathbf{s},\omega)\cdot\boldsymbol{G}^{\dagger}_{\lambda}(\mathbf{s},\mathbf{r}',\omega)=\frac{\hbar\mu_0}{\pi}\omega^2\mathrm{Im}\,\boldsymbol{G}(\mathbf{r},\mathbf{r}',\omega), \tag{15}$$

leads to

$$\left\langle\hat{b}_z(\mathbf{r}_s,t')\hat{b}_z(\mathbf{r}_s,t'')\right\rangle=\int_0^\infty d\omega\,\lambda^2(\omega)\left\{[n_{th}(\omega)+1]e^{-i\omega(t'-t'')}+n_{th}(\omega)e^{i\omega(t'-t'')}\right\}\frac{\hbar\mu_0}{\pi}\hat{z}\cdot\boldsymbol{\nabla}\times\mathrm{Im}\,\boldsymbol{G}(\mathbf{r},\mathbf{r},\omega)\times\overleftarrow{\boldsymbol{\nabla}}\cdot\hat{z}, \tag{16}$$

and hence

$$\begin{aligned}\langle\hat{S}_+\rangle=\rho^{(0)}_{du}\exp&\left[-\int dt'\int dt''\int_0^\infty d\omega\,\lambda^2(\omega)\right.\\
&\left.\times\left\{[n_{th}(\omega)+1]e^{-i\omega(t'-t'')}+n_{th}(\omega)e^{i\omega(t'-t'')}\right\}\frac{\hbar\mu_0}{\pi}\hat{z}\cdot\boldsymbol{\nabla}\times\mathrm{Im}\,\boldsymbol{G}(\mathbf{r},\mathbf{r},\omega)\times\overleftarrow{\boldsymbol{\nabla}}\cdot\hat{z}\right].\end{aligned} \tag{17}$$

The time integrals give

$$\begin{aligned}\int_0^t dt' \int_0^t dt''\, e^{\pm i\omega(t'-t'')} &= \left[\frac{e^{\pm i\omega t}-1}{\pm i\omega}\right]\left[\frac{e^{\mp i\omega t}-1}{\mp i\omega}\right],\\ &= \left[\frac{e^{\pm i\omega t/2}-e^{\mp i\omega t/2}}{\pm i\omega}\right]\left[\frac{e^{\mp i\omega t/2}-e^{\pm i\omega t/2}}{\mp i\omega}\right],\\ &= \left[\frac{\sin(\pm\omega t/2)}{\omega/2}\right]\left[\frac{\sin(\pm\omega t/2)}{\omega/2}\right],\\ &= t^2\left[\frac{\sin(\omega t/2)}{\omega t/2}\right]^2,\\ &= t^2 \operatorname{sinc}^2(\omega t/2)\,. \end{aligned} \tag{18}$$

Thus

$$\langle \hat{S}_+\rangle = \rho_{du}^{(0)} \exp\left[-t^2\int_0^\infty d\omega\, \operatorname{sinc}^2(\omega t/2)\,[2n_{th}(\omega)+1]\,\lambda^2(\omega)\frac{\hbar\mu_0}{\pi}\,\hat{z}\cdot\boldsymbol{\nabla}\times\operatorname{Im}\boldsymbol{G}(\mathbf{r},\mathbf{r},\omega)\times\overleftarrow{\boldsymbol{\nabla}}\cdot\hat{z}\right]. \tag{19}$$

Noting that

$$2n_{th}(\omega)+1 = \coth(\hbar\omega/2k_bT)\,, \tag{20}$$

one arrives at the expression in Eq. 22 in the main text.

Free Space

The free space Greens function reads

$$\boldsymbol{G}^{(0)}(\mathbf{r},\mathbf{r}',\omega) = \left(\boldsymbol{\nabla}\otimes\boldsymbol{\nabla}+\frac{\omega}{c}\mathbb{I}\right)\frac{e^{i\omega|\mathbf{r}-\mathbf{r}'|/c}}{4\pi\omega^2|\mathbf{r}-\mathbf{r}'|/c^2}, \tag{21}$$

We are interested in the $\hat{z}\hat{z}$ component of the curl of the Green's function

$$\hat{z}\cdot\boldsymbol{\nabla}\times\boldsymbol{G}(\mathbf{r},\mathbf{r},\omega)\times\overleftarrow{\boldsymbol{\nabla}}\cdot\hat{z} = \nabla_x G_{yy}(\mathbf{r},\mathbf{r},\omega)\overleftarrow{\nabla}_x + \nabla_y G_{xx}(\mathbf{r},\mathbf{r},\omega)\overleftarrow{\nabla}_y - \nabla_x G_{yx}(\mathbf{r},\mathbf{r},\omega)\overleftarrow{\nabla}_y - \nabla_y G_{xy}(\mathbf{r},\mathbf{r},\omega)\overleftarrow{\nabla}_x. \tag{22}$$

Evaluating this expression leads to

$$\hat{z}\cdot\boldsymbol{\nabla}\times\operatorname{Im}\boldsymbol{G}(\mathbf{r},\mathbf{r},\omega)\times\overleftarrow{\boldsymbol{\nabla}}\cdot\hat{z} = \frac{\omega^3}{6\pi c^3}. \tag{23}$$

Hence

$$\langle \hat{S}_+\rangle = \rho_{du}^{(0)} \exp\left\{-\frac{\hbar\mu_0\gamma^2t^2}{6\pi^2c^3}\int_0^\infty d\omega\,\omega^3 \operatorname{sinc}^2(\omega t/2)\coth(\hbar\omega/2k_bT)\right\}, \tag{24}$$

which is Eq. 24 in the main text.

Homogeneous Media

The homogeneous media Greens function reads

$$\boldsymbol{G}(\mathbf{r},\mathbf{r}',\omega) = \left(\boldsymbol{\nabla}\otimes\boldsymbol{\nabla}+\frac{\omega}{c}n(\omega)^2\mathbb{I}\right)\frac{e^{i\omega n(\omega)|\mathbf{r}-\mathbf{r}'|/c}}{4\pi\omega^2 n(\omega)^2|\mathbf{r}-\mathbf{r}'|/c^2}, \tag{25}$$

Unfortunately, the $\hat{z}\hat{z}$ component of the curl of the Green's function for coincident limits diverges unless the imaginary part of the refractive index, $n(\omega)$, vanishes where upon it equals

$$\hat{z}\cdot\boldsymbol{\nabla}\times\operatorname{Im}\boldsymbol{G}(\mathbf{r},\mathbf{r},\omega)\times\overleftarrow{\boldsymbol{\nabla}}\cdot\hat{z} = \frac{\omega^3}{6\pi c^3}\operatorname{Re}[n(\omega)]^3\,. \tag{26}$$

One can regularize the expression for the coherence by performing local field corrections on the spin. We model the embedding of the spin into the homogeneous medium by applying a real-cavity model, where we assume the spin lies at the centre of a spherical cavity. The Green's function for the field inside a spherical cavity reads

$$\boldsymbol{G}(\mathbf{r},\mathbf{r}',\omega) = \frac{ik}{4\pi}\sum_{n\in e,o}\sum_{l=1}^{\infty}\sum_{m=0}^{l}(2-\delta_{l0})\frac{2l+1}{l(l+1)}\frac{(l-m)!}{(l+m)!} \times\left[R_{TE}(\omega)\boldsymbol{M}_{mln}(k,\mathbf{r})\otimes\boldsymbol{M}_{mln}(k,\mathbf{r}')+R_{TM}(\omega)\boldsymbol{N}_{mln}(k,\mathbf{r})\otimes\boldsymbol{N}_{mln}(k,\mathbf{r}')\right], \quad (27)$$

where R_{TE} and R_{TM} are the reflection coefficients for the TE and TM polarized waves respectively and the $\boldsymbol{M}_{mln}(k,\mathbf{r})$ and $\boldsymbol{N}_{mln}(k,\mathbf{r})$ dyads are given by

$$\boldsymbol{M}_{ml\binom{e}{o}}(k,\mathbf{r}) = \mp\frac{m}{\sin\theta}j_l(kr)P_l^m(\cos\theta)\begin{pmatrix}\sin m\phi\\ \cos m\phi\end{pmatrix}\underline{e}_\theta - j_l(kr)\frac{dP_l^m(\cos\theta)}{d\theta}\begin{pmatrix}\cos m\phi\\ \sin m\phi\end{pmatrix}\underline{e}_\phi, \quad (28)$$

$$\boldsymbol{N}_{ml\binom{e}{o}}(k,\mathbf{r}) = \frac{l(l+1)}{kr}j_l(kr)P_l^m(\cos\theta)\begin{pmatrix}\cos m\phi\\ \sin m\phi\end{pmatrix}\underline{e}_r + \frac{1}{kr}\frac{d}{dr}[rj_l(kr)]\left[\frac{dP_l^m(\cos\theta)}{d\theta}\begin{pmatrix}\cos m\phi\\ \sin m\phi\end{pmatrix}\underline{e}_\theta \mp\frac{m}{\sin\theta}P_l^m(\cos\theta)\begin{pmatrix}\sin m\phi\\ \cos m\phi\end{pmatrix}\underline{e}_\phi\right], \quad (29)$$

where $j_l(x)$ are spherical Bessel functions of the first kind and $P_l^m(x)$ are the associated Legendre polynomials. One can compute the curl of the individual dyads using

$$\boldsymbol{\nabla}\times\mathbf{A} = \frac{1}{r\sin\theta}\left(\frac{\partial}{\partial\theta}(A_\phi\sin\theta)-\frac{\partial A_\theta}{\partial\phi}\right)\underline{e}_r + \frac{1}{r}\left(\frac{1}{\sin\theta}\frac{\partial A_r}{\partial\phi}-\frac{\partial}{\partial r}(rA_\phi)\right)\underline{e}_\theta + \frac{1}{r}\left(\frac{\partial}{\partial r}(rA_\theta)-\frac{\partial A_r}{\partial\theta}\right), \quad (30)$$

then by taking $\mathbf{r},\mathbf{r}'\to 0$ one finds that the only contribution is from $l=1$ and $m=0$. Hence, the TM mode vanishes and only the TE mode contributes. Thus, the Green's function reduces to

$$\hat{z}\cdot\boldsymbol{\nabla}\times\boldsymbol{G}(\mathbf{r},\mathbf{r}',\omega)\times\overleftarrow{\boldsymbol{\nabla}}\cdot\hat{z}\Big|_{\mathbf{r},\mathbf{r}'\to 0} = \frac{3ik}{8\pi}R_{TE}(\omega)\sum_{n\in e,o}\boldsymbol{\nabla}\times\boldsymbol{M}_{01n}(k,\mathbf{r})\otimes\boldsymbol{M}_{01n}(k,\mathbf{r}')\times\overleftarrow{\boldsymbol{\nabla}}\Big|_{\mathbf{r},\mathbf{r}'\to 0} = i\frac{\omega^3}{6\pi c^3}R_{TE}(\omega). \quad (31)$$

The reflection of the TE modes at the cavity interface can be described in terms of the Mie scattering coefficient

$$R_{TE}(\omega) = \frac{h_1^{(1)}(z_0)\left[zh_1^{(1)}(z)\right]'-\mu(\omega)h_1^{(1)}(z)\left[z_0h_1^{(1)}(z_0)\right]'}{\mu(\omega)h_1^{(1)}(z)\left[z_0j_1^{(1)}(z_0)\right]'-j_1^{(1)}(z_0)\left[zh_1^{(1)}(z)\right]'} \quad (32)$$

where $z_0=\omega R_c/c$ and $z=n(\omega)\omega R_c/c$, with R_c the radius of the cavity, and $j_1(z)$ and $h_1(z)$ are, respectively, spherical Bessel and Hankel functions of the first kind for $l=1$,

$$j_1(z) = \frac{\sin(z)}{z^2}-\frac{\cos(z)}{z}, \quad h_1(z) = \left(\frac{1}{z}+\frac{i}{z^2}\right)e^{iz}. \quad (33)$$

We will assume that R_c is small compared to the main wavelengths associated with the decoherence process and expand $R_s(\omega)$ in powers of $\omega R_c/c$

$$R_{TE}(\omega) = \frac{3\mu(\omega)-3}{[2\mu(\omega)+1]}\frac{ic^3}{\omega^3R_c^3}+\frac{9}{5}\left\{\frac{\mu(\omega)^2[5\varepsilon(\omega)-1]-3\mu(\omega)-1]}{[2\mu(\omega)+1]^2}\right\}\frac{ic}{\omega R_c}-9\frac{\mu(\omega)^{5/2}\varepsilon(\omega)^{3/2}}{[2\mu(\omega)+1]^2}+1+\mathscr{O}\left(\frac{\omega R_c}{c}\right). \quad (34)$$

Thus, to leading order we have

$$\hat{z}\cdot\boldsymbol{\nabla}\times\boldsymbol{G}(\mathbf{r},\mathbf{r}',\omega)\times\overleftarrow{\boldsymbol{\nabla}}\cdot\hat{z} = i\frac{\omega^3}{6\pi c^3}\frac{3\mu(\omega)-3}{[2\mu(\omega)+1]}\frac{ic^3}{\omega^3R_c^3}+\mathscr{O}\left(\frac{\omega R_c}{c}\right), = -\frac{1}{2\pi R_c^3}\frac{\mu(\omega)-1}{[2\mu(\omega)+1]}+\mathscr{O}\left(\frac{\omega R_c}{c}\right). \quad (35)$$

Taking the imaginary part of the double curl of the Green's function leads to

$$\hat{z}\cdot\boldsymbol{\nabla}\times\mathrm{Im}\,\boldsymbol{G}(\mathbf{r},\mathbf{r}',\omega)\times\overleftarrow{\boldsymbol{\nabla}}\cdot\hat{z} = \frac{3}{2\pi R_c^3}\frac{-\mathrm{Im}\,\mu(\omega)}{4[\mathrm{Im}\,\mu(\omega)]^2+[1+2\mathrm{Re}\,\mu(\omega)]^2}. \quad (36)$$

Substituting this back into the expression for the coherence gives

$$\langle \hat{S}_+ \rangle = \rho_{du}^{(0)} \exp\left[-\frac{3\hbar\mu_0\gamma^2 t^2}{2\pi^2 R_c^3} \int_0^\infty d\omega \,\mathrm{sinc}^2\left(\omega t/2\right) \coth\left(\hbar\omega/2k_bT\right) \frac{-\mathrm{Im}\,\mu(\omega)}{4[\mathrm{Im}\,\mu(\omega)]^2 + [1+2\mathrm{Re}\,\mu(\omega)]^2} \right], \tag{37}$$

which is Eq. 33 in the main text.
